# Early Disseminated Lyme Disease with Carditis Complicated by Posttreatment Lyme Disease Syndrome

**DOI:** 10.1155/2017/5847156

**Published:** 2017-10-31

**Authors:** Cheryl Novak, Andrew Harrison, John Aucott

**Affiliations:** ^1^Division of Rheumatology, Department of Medicine, Johns Hopkins University School of Medicine, 2360 W. Joppa Road, Suite 320, Lutherville, MD 21063, USA; ^2^Medical Laboratory Sciences Program, Quinnipiac University, 275 Mount Carmel Avenue, Hamden, CT 06518, USA

## Abstract

Lyme disease is an infectious disease caused by the bacterium *Borrelia burgdorferi*. When untreated, infection may spread to the heart, nervous system, and joints. Cardiac involvement usually manifests as abnormalities of the conduction system and bradycardia. Treatment of Lyme disease is generally effective, with a subset of patients experiencing persistent, sometimes long-term symptoms called posttreatment Lyme disease syndrome.

## 1. Background

Lyme disease is a multistage infectious disease caused by infection with the spirochete bacteria *Borrelia burgdorferi* [1]. Early Lyme disease is characterized by the hallmark skin lesion called erythema migrans (EM) [1]. In approximately 20% of patients, the EM rash may not be present or may not be recognized [2]. Without the typical skin lesion, the signs and symptoms of Lyme disease overlap with a nonspecific viral infection that includes fatigue, fever, chills, and malaise.

If untreated, *B. burgdorferi* may disseminate through the bloodstream resulting in joint, neurologic, and cardiac involvement. Objective manifestations of early dissemination may include multiple skin lesions, joint synovitis, meningitis, or carditis [1]. However, in the absence of objective organ system involvement, a subset of patients may only present with fatigue, sweats, malaise, and arthralgia [3].

The prognosis of Lyme disease is generally very good, especially when treated in the localized EM stage. Posttreatment Lyme disease syndrome (PTLDS) may occur after antibiotic treatment of any stage of Lyme disease and is associated with ongoing symptoms of fatigue, musculoskeletal pain, and cognitive complaints. These symptoms may wax, wane, and persist for years and may be mild or, in other cases, can result in a significant decline in health-related quality of life [4]. The known risk factors for PTLDS include greater severity of initial disease, presence of initial neurologic involvement, delay in diagnosis and treatment, and the persistence of symptoms after the completion of initial antibiotic therapy [5, 6]. The pathophysiology of and effective therapy for PTLDS is the subject of ongoing research [7, 8].

## 2. Case Presentation

**Figure 1:**
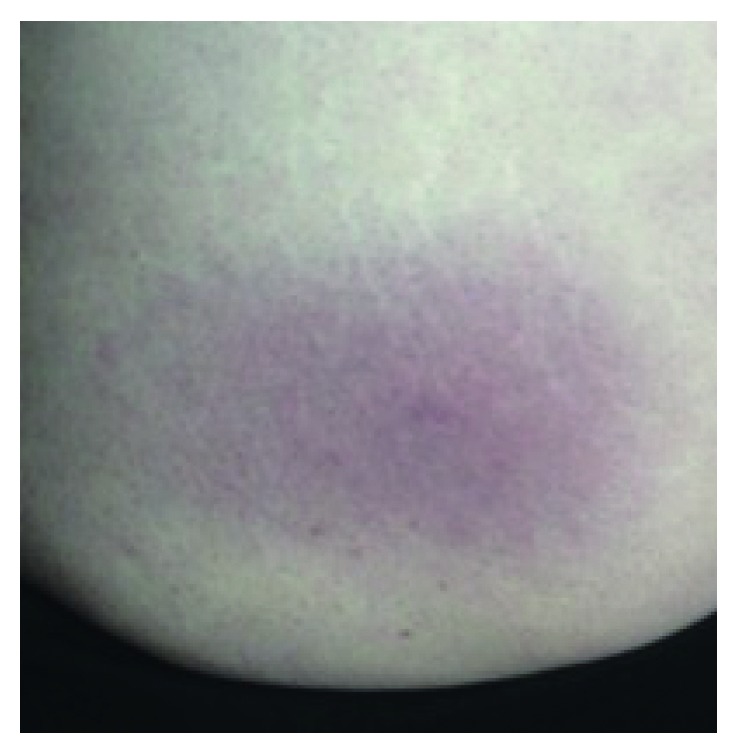
The patient noted an oval nonpainful red skin lesion on her left hip which was attributed to a drug reaction.

A previously healthy 48-year-old woman living in rural Maryland presented in early June with a two-week history of neck pain, fatigue, anorexia, nausea, and intermittent fevers and chills. She was concerned that she might have Lyme disease because of tick bites in the past and ongoing recreational exposure to ticks but did not recall a tick bite in the weeks preceding the onset of her illness. A tentative diagnosis of a viral illness, possibly mononucleosis, was made during an initial visit to her primary care physician. She presented to the emergency room the following day with worsening neck pain, stiffness, and myalgia and was admitted for dehydration and further evaluation. Physical exam showed a temperature of 101.1°F, pulse 101 beats per minute, and a new oval nonpainful red rash on her left hip which was attributed to a drug reaction (Figure 1). Laboratory evaluation of complete blood count (CBC) showed a total white blood cell (WBC) count of 4.9·10^3^ U/L with 8% lymphocytes and a low absolute lymphocyte count of 400 U/L. A comprehensive metabolic panel (CMP) showed an abnormal AST of 245 U/L, ALT of 218 U/L, and alkaline phosphatase of 215 U/L. A CT scan of the head without contrast was negative. A lumbar puncture showed one white blood cell, normal protein, a normal glucose level, negative bacterial culture, and negative Lyme antibodies in the CSF. Her blood cultures, hepatitis serology, and influenza A and B were also negative. The Epstein-Barr virus serology was interpreted as consistent with either late primary infection or reactivation of mononucleosis with a (+) IgG antibody, (−) IgM, and (+) EBNA. She was discharged with a diagnosis of mononucleosis due to EBV and recommendations for symptomatic care. A Lyme serology was sent with pending results at the time of discharge that subsequently showed no ELISA done, (+) IgM Western blot, and (−) IgG Western blot.

Over the next 4 weeks, she had waxing and waning symptoms of fever, fatigue, arthralgia, and anorexia. She developed new complaints of difficulty finding words and trouble concentrating, especially with reading. The original skin lesion on her hip enlarged, and new skin lesions appeared on her left lower leg and stomach. She was treated with Keflex for a presumed bacterial cellulitis. The rash did not respond to the antibiotics but eventually resolved over a period of weeks.

**Figure 2:**
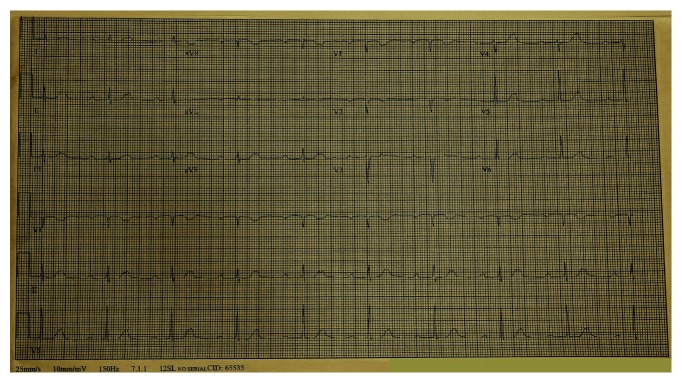
The patient's admitting EKG showing sinus tachycardia with second-degree AV block with 2 : 1 conduction.

Over the next week, she developed increasing exertional dyspnea when climbing stairs, anxiety, and chest heaviness. The patient also noted an irregular heart beat and that, on one occasion, her pulse rate was 31 bpm. She felt like she was going to pass out and returned to the emergency department 6 weeks after her initial visit for further evaluation. There, she was noted to be bradycardic with a pulse rate in the 40's. The admitting EKG showed sinus tachycardia with new onset second-degree AV block with 2 : 1 conduction (Figure 2). The chest X-ray was normal, and a transthoracic echocardiogram did not show any valvular or structural abnormalities, with an ejection fraction of sixty percent. Her repeat Lyme ELISA was positive, and WB came back with all three IgM bands positive, and IgG was positive for eight out of ten bands, confirming the diagnosis of Lyme disease with carditis. The AST and ALT were normal at 29 U/L and 44 U/L, respectively. Following treatment with three doses of ceftriaxone, the AV block resolved while in the hospital, and she was discharged on a 3-week course of doxycycline.

After treatment with 21 days of doxycycline, the patient had resolution of her shortness of breath and palpitations and had gradual improvement in her symptoms of fever, headache, and anorexia. She remained fatigued and had increasing problems with insomnia. There was also ongoing trouble with word finding, focusing, and concentrating. A stress test showed above-average exercise capacity with no arrhythmia. She developed new symptoms of bursitis in hips, coldness in hands, shooting pains in arms, and numbness in hands and feet. She stated that she “feels like a 90-year-old woman” with hip and knee pain and remained out of work from July to September. Ten weeks after completion of her doxycycline, an infectious disease consultation was obtained for evaluation of her ongoing fatigue, hip pain, paresthesias, and difficulty with concentration. The consult felt as though the original EBV serologies were consistent with remote exposure and not an acute infection. Additionally, the patient's ongoing symptoms following successful treatment of her Lyme carditis were due to slowly resolving symptoms from her previously treated Lyme disease. A repeat convalescent Lyme serology performed by Quest diagnostics was negative, with a positive ELISA and IgM western blot, but a negative IgG western blot with four bands. The CBC, CMP, sedimentation rate, and C-reactive protein were normal.

Seven months after completion of the antibiotic treatment for her Lyme carditis, the patient sought a second opinion for her continued symptoms and resulting substantial reduction in her ability to function at work and home. The patient had no evidence of other tick-borne infection or other medical causes of her fatigue and musculoskeletal pain. Based on the Infectious Diseases Society of America case definition, she was diagnosed with posttreatment Lyme disease syndrome. Eighteen months after resolution of her Lyme carditis, she is working full time and continues to report her pain and fatigue levels as a 4/10, which she rates as mildly disrupting her work, social, and family life.

## 3. Discussion

Posttreatment Lyme disease syndrome is a well-described constellation of symptoms that may follow the treatment of any stage of Lyme disease [1]. The illness is characterized by symptoms of fatigue, musculoskeletal pain, and cognitive complaints that persist for at least 6 months after completion of standard antibiotic therapy of well-documented Lyme disease [9]. As our case illustrates, complex symptoms such as shooting pains, numbness, and tingling may also occur. A published case definition of PTLDS by the Infectious Diseases Society of America proposes that the symptoms should be of significant severity such that they impact daily functioning [9]. As was the case for our patient, symptoms may be severe for months, resulting in loss of work and significant social and personal loss in quality of life. Our patient's manifestations have persisted for over 18 months, which is consistent with other reports of chronic symptoms lasting over a decade, especially in patients with disseminated infection and a delay in initial antibiotic therapy [10].

The diagnosis of PTLDS is made by recognizing the onset of symptoms of fatigue, pain, and cognitive difficulties following a documented case of appropriately treated Lyme disease. Persisting symptoms for 6 months or longer that interfere with quality of life are consistent with PTLDS [9]. Serology at the time of the PTLDS diagnosis may be negative, as immune memory for remote infection wanes over time after treatment [11]. Because serology is not diagnostic for PTLDS, other conditions that may explain the patient's symptoms must be excluded by a complete evaluation.

Risk factors for PTLDS include greater severity of initial disease prior to treatment of Lyme disease, delay in diagnosis, and the presence of neurologic manifestations [6]. Our patient is one of the first reported cases of PTLDS following Lyme carditis. The patient's carditis was well documented with a presentation of heart block that resolved quickly after the administration of appropriate antibiotics. In a prospective study of patients with EM who were not treated, 1% of patients developed carditis with evidence of AV nodal block [12]. Clinical symptoms that are most commonly reported with Lyme carditis other than conduction disturbances are lightheadedness, palpitations, shortness of breath, chest pain, and syncope. The manifestations of carditis, including heart block, respond quickly to antibiotic therapy. The development of carditis is hypothesized to be a risk factor for PTLDS because it ensues during the disseminated phase of infection, which is more likely to occur when there is a delay in diagnosis. This expectation is consistent with other recently reported cases of carditis, in which a delay in diagnosis resulted in death [12, 13].

The delayed diagnosis in our patient points out several pitfalls in the diagnosis of early Lyme disease. The EM rash is a single or multiple expanding round or oval lesion that does not have the typical diffuse maculopapular rash seen in EBV or viral exanthems. The diagnosis is challenging when the diagnostic rash either does not develop or lacks the stereotypical bull's eye appearance and is misattributed to other causes, such as a drug reaction as was seen in our patient. In these instances, which account for approximately 20% of cases, flu-like symptoms may be attributed to a viral illness [14].

One often overlooked clue in the diagnosis of atypical presentations of early Lyme disease are seasonal and geographic hints in the presenting history. For example, viral respiratory illnesses concentrate in the winter months, while tick-borne illnesses occur primarily in the warm months of the year [15]. Geographic risk factors are especially important in consideration of zoonosis and vector-borne infections where the geographic resident or travel history often points to a specific differential diagnosis. In the United States, Lyme disease is highly endemic in the Great Lakes region and the Northeast and Mid-Atlantic States.

Laboratory findings, especially CBC results, can be helpful in narrowing the diagnosis of viral-like symptoms. In the case presented, there should have been a degree of caution in attributing the patient's initial illness to EBV, given the lack of lymphocytosis and absence of atypical lymphocytes. While false-positivity can occur with Lyme disease assays when a patient has an infection with EBV, in retrospect, this patient did not have evidence of acute EBV infection. Conversely, a recent report documents two cases where early disseminated Lyme disease caused false-positive serology for EBV infection [16]. The presence of a normal total WBC count is typical for Lyme disease, a distinction from other bacterial infections where leukocytosis and increased polymorphonuclear cells are characteristically seen [17]. However, prolonged nonrespiratory symptoms are atypical in uncomplicated viral infections and, in the appropriate geographic setting, should suggest Lyme disease.
